# 肺部疾病介入诊疗技术的现状及发展

**DOI:** 10.3779/j.issn.1009-3419.2020.102.09

**Published:** 2020-06-20

**Authors:** 金明 徐, 舟 安, 哲浩 何, 望 吕, 坚 胡

**Affiliations:** 310003 杭州，浙江大学医学院附属第一医院胸外科 Department of Thoracic Surgery, the First Affiliated Hospital of Zhejiang University, Zhejiang University School of Medicine, Hangzhou 310003, China

**Keywords:** 肺部疾病, 介入技术, 发展现状, Pulmonary disease, Interventional technology, Development status

## Abstract

以支气管镜为核心的肺部疾病介入诊疗技术经历了百余年的发展历史，逐渐形成了多个临床学科合作配合，多种介入微创技术深度整合，多样化诊疗理念交叉融合的肺部疾病诊疗模式。本文对肺部疾病介入诊疗技术的现状及发展作一综述，同时介绍本中心介入诊疗特色。

## Abstract (en)

Bronchoscope is the core part of the interventional diagnosis and treatment technology for lung diseases, which has experienced more than 100 years of development history. It has gradually formed a diagnosis and treatment model of pulmonary diseases with cooperation of multi-clinical disciplines, deep integration of interventional minimally invasive techniques, and cross-integration of diversified diagnosis and treatment concepts. This article aimed to review the current status and development of interventional diagnosis and treatment techniques for lung diseases, and introduces the characteristics of the interventional treatment of our center.

## 1 肺部疾病介入诊疗技术的发展历程

科学技术的进步给疾病的诊疗模式带来了革命性的进展，在120余年的发展史中，介入诊疗技术在肺部疾病中的地位越来越重要，从传统硬质支气管镜到纤维支气管镜，再到现代的电子支气管镜、纤维支气管镜、电视硬支气管镜同台竞技的时代^[1]^，人类借助器械对肺部结构的探索从未停止，以支气管镜为核心的介入技术实现了经自然腔道肺部疾病诊治新模式。

### 1.1 支气管镜的发展历程

从1897年至今，支气管镜的发展经历了诸多变革（如表 1所示），历史上诸位医学大家不断的赋予了支气管镜新的定义和功能。借助于纤维支气管镜，人类第一次完整的观察到了支气管树的腔内结构，对各级支气管进行了重新命名，并于1972年出版了纤维支气管镜图谱^[2]^。纤维支气管镜在肺部疾病的诊断，尤其是中央型肺癌的诊断中发挥了决定性的作用。随后，电子支气管镜的发明大大提高了成像系统的清晰度，硬质支气管镜下的介入操作在麻醉技术安全性提高的保障下在近10年来焕发了新的生命力。

**1:** 支气管镜的发展历程 Development process of bronchoscope

Time	Personage	Event	Remarks
1897	Gustav Killian^[11]^	Father of bronchoscope	First report of using esophagoscope to remove airway foreign body
1899	Chevalier Jackson^[12]^	Father of tracheoesophagology	Improvement of traditional rigid bronchoscope and establishment of standardized operation procedure
1950s	Broyles *et al*^[13]^	Birth of therapeutic tracheoscope	Improve lens and rotation angle
1965	Anderson *et al*^[14]^	First bronchoscopic lung biopsy	Diagnosis of metastatic adenocarcinoma by rigid bronchoscope
1968	Shigeto Ikeda^[15]^	Birth of fibrobronchoscope	Milestones in the history of bronchoscope
1970	Shigeto Ikeda	Olympus promoted the production of fibrobronchoscope, and the atlas of fiberoptic bronchoscope came out	
1974	Shigeto Ikeda	World Association of Bronchus, First Congress of Bronchus	
1983	Welch Allyn company^[16]^	Electronic camera endoscopy	Ashahi-Pentax then launched an electronic bronchoscope

### 1.2 超声支气管镜的发展历程

超声技术应用于支气管镜使得经腔内探测纵隔及肺门淋巴结成为可能，超声支气管镜（endobroncheal ultrasonography, EBUS）引导下的针吸活检术（transbronchial needle aspiration, TBNA）和内镜超声引导细针抽吸术（endoscopic ultrasound-guided fine needle aspiration, EUS-FNA）已经广泛开展，联合采用EBUS-TBNA和EUS-FNA可提高肺门及纵隔淋巴结术前分期的准确性^[3, 4]^。美国胸外科医师学会（American College of Chest Physicians, ACCP）指南推荐为非小细胞肺癌的侵入性分期方法更加倾向于采用EBUS-TBNA^[5]^。此外，EBUS-TBNA技术可用于立体定向放射治疗的淋巴结转移评估^[6]^，进一步联合快速现场细胞学评估（rapid on-site cytologic evaluation, ROSE）技术对小细胞肺癌同样具有较高的诊断效能^[7]^。烧灼辅助支气管钳活检（cautery-assisted transbronchial forceps biopsies, ca-TBFB）较传统EBUS-TBNA相比可获取更大的组织块^[8]^。近年来，自发荧光、窄带成像、高倍成像等技术的应用提高了支气管镜的诊断精度^[9]^。射线支气管内超声（radial endobronchial ultrasound, R-EBUS）、光学相干断层扫描（confocal laser endomicroscopy, OCT）、共焦激光显微内窥镜（confocal laser endomicroscopy, CLE）和激光拉曼光谱（laser Raman spectroscopy, LRS）等新技术的发展进一步推动了支气管镜技术的进步^[9]^。

### 1.3 介入肺脏病学的创立

2002年，欧洲呼吸病学会（European Respiratory Society, ERS）和美国胸科学会（American Thoracic Society, ATS）提出了“介入肺脏病学”的概念^[10]^，将其定义为：“是一门涉及呼吸病侵入性诊断和治疗操作的医学科学和艺术”。该学科以支气管镜为主要工具，包括胸外科、呼吸科、放射科、麻醉科、手术室团队在内的临床多学科合作诊疗模式逐渐形成。

### 1.4 电磁导航支气管镜的发展历程

低剂量薄层CT广泛应用使得越来越多的早期肺癌被检出^[17]^，面对周围型孤立性肺小结节，传统纤维支气管镜无法到达，电磁导航支气管镜技术（electromagnetic navigation bronchoscopy, ENB）的出现使得外周病灶的定位和活检成为可能^[18-21]^，且具有并发症少、经自然腔道获取病理、创伤小等优势^[22-24]^。目前ENB辅助引导下可实现经支气管肺活检，经支气管淋巴结针吸活检，与支气管超声实时定位联合应用，与PET-CT及ROSE技术联合应用，具有较高的安全性和有效性。更进一步的，ENB引导下通过荧光和放射性造影剂实现小结节定位为电视辅助胸腔镜手术（video-assisted thoracoscopic surgery, VATS）精准切除病灶提供了新的术中定位模式，实现了介入技术与微创技术的融合；ENB为立体放疗基准粒子放置的定位提供了安全有效的新方法，ENB引导下的气道内近距离放疗为无法手术的肺外周肿瘤提供了新的思路，实现了介入技术与放疗技术的融合。ENB引导经支气管镜射频消融治疗和微波治疗实现了经自然腔道肿瘤定点清除，为无法耐受手术患者提供了微创诊疗选择。

### 1.5 经支气管镜介入治疗的发展

经支气管介入治疗在中央型气道梗阻（central airway obstruction, CAO）的治疗中有重要地位，包括气道扩张、支气管镜下消融治疗、支气管内支架置入、局部化疗药注射、支气管腔内放疗等^[25-28]^。其中，现代气道内支架的发展始于1987年，经历了30余年的发展，气道支架的材质、形状、长度及患者预后都有了很大的改善^[29, 30]^。气道支架根据材质可分为金属支架和非金属支架，根据有无被膜，金属支架可分为被膜支架和裸支架，非金属支架可分为硅酮支架和塑料支架等。主要应用于恶性中心气道狭窄的管腔重建，局部支气管管腔或瘘口的封堵等。近年来，随着3D打印技术的发展，三维重建气道模型对于气道狭窄的治疗提供了术前规划、演练和教学功能^[31]^。

支气管镜下治疗手段的能量来源非常多样化，主要包括高频电刀、氩等离子体凝固（新一代高频电刀）^[32]^、激光（YAG, Nd:YAG）^[33]^、微波、CO_2_冷冻^[34]^，光动力治疗^[35]^等多种方法。各种镜下治疗方法有共性也有个性，可相互补充，为支气管镜下的治疗手段革新提供了广阔的思路。

## 2 浙江大学医学院附属第一医院胸外科介入诊疗特色

本中心是拥有介入诊疗技术的胸外科单位，实现了介入技术与微创技术的杂交应用和深度融合，为患者提供一站式诊疗，全面参与各种阶段和病情的肺部疾病的诊治。常规开展EBUS-TBNA、微波、射频、粒子、单向活瓣、硬质气管镜、支架、ENB等介入诊疗技术。

本中心常规开展EBUS-TBNA纵隔及肺门淋巴结微创活检术，近3年来累计789例，并创新性融合弹性超声技术^[36]^，通过超声的蓝色占比（blue color proportion, BCP）半定量指数预测淋巴结的良恶性，AUC值达到0.86（95%CI: 0.78-0.94），辅助临床医生对淋巴结良恶性进行判定，具有较高的安全性和诊断准确率。同时，本中心开展各种复杂气道支架植入术，同时对气道支架在晚期气道恶性病变患者中的临床效果及并发症做了系统的评估并对潜在的危险因素进行了分析，有助于改善气道支架的临床应用效果^[37]^，我们也成功开展过ECMO支持下的危重患者气道支架植入术，挽救了患者的生命。

本中心也是国内率先开展电磁导航支气管镜技术的单位之一（图 1），经支气管自然腔道获取病理组织，精准诊断肺部小结节，尤其是对周围型小结节的诊断相比传统气管镜具有明显优势，避免了在CT引导下行有创性经皮穿刺活检。同时，ENB结合ROSE技术实现快速诊断，可与微创手术融合，手术室场景下完成疾病的“诊断、定位、外科切除”一体化诊疗模式。截止2019年10月，本中心ENB已完成352余例，位居国内首位。

**1:**
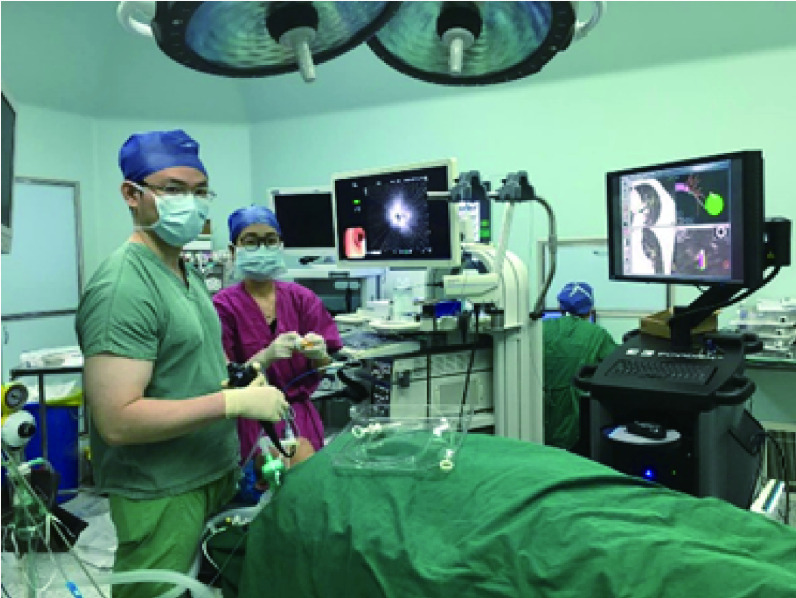
手术室场景下开展电磁导航支气管镜技术实现肺结节的精准诊断 Accurate diagnosis of pulmonary nodules by electromagnetic navigation bronchoscopy in the operating room

手术室场景下由外科医生进行介入操作可以为患者提供强有力的安全保证，在面对出血、氧合下降等紧急情况时，外科团队、麻醉团队、手术室护理团队及齐全的抢救设备可以为患者保驾护航，大大提高了介入操作的安全性和可行性。
